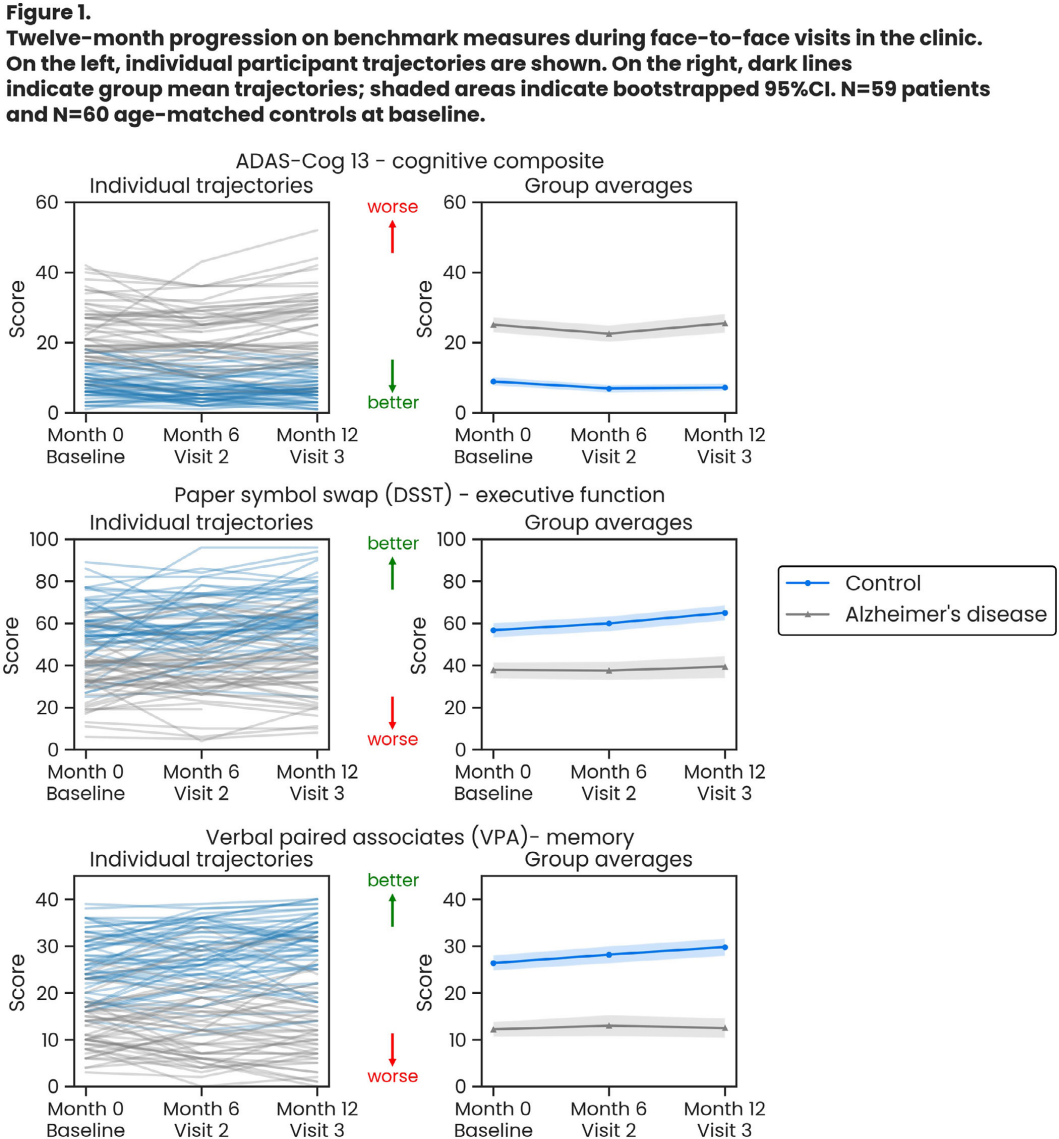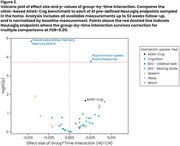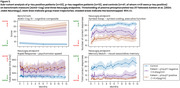# Frequent at‐home multimodal measurements are more sensitive to progression than the gold‐standard clinic‐based ADAS‐Cog composite scale

**DOI:** 10.1002/alz70863_110568

**Published:** 2025-12-23

**Authors:** Laura M Rueda‐Delgado, Florentine M Barbey, Alison R Buick, Shannon Diggin, John Dyer, Hugh Nolan, James B Rowe, Brian Murphy

**Affiliations:** ^1^ Cumulus Neuroscience, Dublin Ireland; ^2^ Cumulus Neuroscience, Belfast UK; ^3^ Department of Clinical Neurosciences, University of Cambridge, Cambridge, Cambridgeshire UK

## Abstract

**Background:**

The detection of progression is key to the statistical power of placebo‐controlled clinical trials. This is a challenge in dementia, as disease progression is confounded with natural aging, and as widely used assessments (CDR, ADAS‐Cog) lack sensitivity and are administered only infrequently and in specialist settings.

We report a non‐interventional study conducted with 10 pharma companies to evaluate the feasibility and value of capturing repeated multimodal digital assessments with Alzheimer's dementia patients, using the NeuLogiq^TM^ platform in the home over 12 months. The difference in progression between mild dementia and matched controls serves as a model of placebo vs. an efficacious intervention. We quantify the statistical power of NeuLogiq digital measures, relative to conventional endpoints.

**Method:**

Seven UK sites recruited Alzheimer's type mild dementia patients (*n* = 59, ACE‐III scores >60 and ≤88) and controls (*n* = 60). Participants were asked to perform 8 cognitive tasks presented on a tablet with synchronous low‐burden EEG, autonomously at home. Overnight EEG was also recorded. NeuLogiq sessions were staggered throughout the year, with 26 samples requested per task. Benchmark paper‐based assessments (including ADAS‐Cog) were administered in‐clinic (months 0, 6, 12) and blood plasma was collected (months 6, 12). Progression was analyzed with mixed effects models, for benchmarks and a predefined selection of Cumulus variables.

**Result:**

Over 52 weeks, there was a real but modest difference in progression on ADAS‐Cog (beta=2.03, *p* = 0.021) and DSST (beta=‐5.67, *p* = 0.006), and no significant difference on VPA, with all showing familiarization effects (Fig. 1). Of the 41 predefined NeuLogiq measures, several exhibited a similar/larger effect size to ADAS‐Cog (Fig. 2). Two survived correction for multiple comparisons: tablet‐based associative memory task (beta=‐4.52, *p* <0.001) and psychomotor task (beta= 42.4, *p* <0.001). Post‐hoc analysis showed that *p* ‐tau positive/negative patients were separated on the memory task, and on the symbol coding executive function task (Fig. 3).

**Conclusion:**

Individual tablet‐based tasks, completed autonomously at home by mild dementia patients, are more sensitive measurements of disease progression than ADAS‐Cog. In on‐going analysis we are quantifying the additional statistical power that multimodal/multivariate/composite measures may provide, and the resulting potential reductions in size, duration and risk of clinical trials in dementia.